# Extracellular vesicle-mediated immune crosstalk in rheumatoid arthritis synovium: mechanistic insights and translational challenges

**DOI:** 10.3389/fimmu.2026.1891984

**Published:** 2026-07-30

**Authors:** Feng Luo, Xuemei Yuan, Heng Zhou, Qiuyi Wang, Changming Chen, Yi Ling, Jiasi Zheng, Xueming Yao, Wukai Ma

**Affiliations:** 1 Guizhou University of Traditional Chinese Medicine, Guiyang, China; 2 Department of Rheumatology and Immunology, Second Affiliated Hospital of Guizhou University of Traditional Chinese Medicine, Guiyang, China; 3 Department of Traditional Chinese Medicine, The Second People’s Hospital of Guizhou Province, Guiyang, China

**Keywords:** autoimmunity, biomarkers, extracellular vesicles, fibroblast-like synoviocytes, macrophages, rheumatoid arthritis, small extracellular vesicles, synovial microenvironment

## Abstract

Rheumatoid arthritis (RA) is a chronic systemic autoimmune disease marked by persistent synovial inflammation, autoantibody production, cartilage damage, and bone erosion. Biologic disease-modifying antirheumatic drugs (DMARDs) and targeted small-molecule therapies have improved disease control, but durable remission remains difficult for many patients. Low-grade synovitis and structural damage may continue despite treatment. Extracellular vesicles (EVs) may contribute to this residual activity by carrying signals that are not captured by soluble cytokine measurements alone. In the inflamed joint, EVs are released by fibroblast-like synoviocytes, macrophages, neutrophils, endothelial cells, chondrocytes, and osteoclast precursors. Their cargo includes citrullinated proteins, inflammatory mediators, miRNAs, lipids, and matrix-degrading enzymes and often reflects the state of the parent cell. This mini-review examines EV biogenesis, movement across the synovial barrier, and uptake by recipient cells in RA. It evaluates evidence linking EV cargo to NF-κB, MAPK, JAK-STAT, and cGAS-STING signaling; innate and adaptive immune activation; fibroblast-like synoviocyte invasion; and osteoclast differentiation. We also assess the current evidence for EVs as biomarkers and therapeutic vehicles, with particular attention to methodological and translational limitations.

## 1 Introduction

Rheumatoid arthritis (RA) is a chronic systemic autoimmune disease in which synovitis, autoantibody production, cartilage erosion, and bone destruction develop together (1). Treatment options have expanded considerably over the past two decades. Conventional disease-modifying antirheumatic drugs (DMARDs) remain the foundation of therapy, and biologic or targeted synthetic agents can inhibit TNF and IL-6 signaling, T-cell co-stimulation, B-cell activity, and JAK-dependent pathways. Even with these options, sustained remission is not achieved in a substantial proportion of patients (2, 3). The clinical picture is also uneven. Radiographic damage can progress despite apparently adequate cytokine blockade, and ultrasound may detect low-grade synovitis when serum CRP is within the reference range (4). These observations are difficult to reconcile with a model in which one dominant inflammatory pathway accounts for all ongoing disease activity.

Extracellular vesicles (EVs) offer one way to examine residual disease activity outside conventional soluble cytokine networks. Following MISEV2023, we use operational terms such as small extracellular vesicles (sEVs, <200 nm) and reserve biogenesis-based labels for studies that provide the required experimental evidence (5). Where the cited literature uses them, microvesicles refer to plasma membrane-derived vesicles commonly measuring about 200 nm to 1 μm, and apoptotic bodies to larger vesicles often exceeding 1 μm. EVs contain proteins, nucleic acids, lipids, and post-translationally modified antigens. Their composition often mirrors the transcriptional, metabolic, and activation state of the source cell rather than representing membrane turnover alone. In inflamed synovium, those source cells include fibroblast-like synoviocytes (FLS), other stromal cells, macrophages, neutrophils, T and B cells, vascular cells, platelets, chondrocytes, and osteoclast precursors (6, 7). EVs released from these compartments can therefore carry inflammatory and tissue-remodeling signals across the joint.

The relevance of EV biology in RA therefore lies not merely in the presence of vesicles in inflamed joints, but in their capacity to shape the direction and persistence of intercellular communication. Pharmacological blockade of TNF or IL-6 can suppress major soluble inflammatory pathways, but stromal- and immune-cell-derived sEVs may continue to relay activating signals between synovial cell populations independently of soluble cytokines. FLS-derived sEVs have been reported to promote macrophage migration (8). Separately, miRNAs that are dysregulated in RA synovial tissue and synovial-fluid EVs, including miR-155, are linked to NF-κB signaling and macrophage polarization (9–12). Consistent with such reciprocal signaling, macrophage-derived sEVs from arthritic mice have been shown to drive RA-FLS proliferation and inflammatory responses through transfer of miRNA cargo (13). On this basis, it has been hypothesized that FLS-derived sEVs promote a pro-inflammatory macrophage phenotype through NF-κB-activating cargo, and that macrophage-derived sEVs reciprocally reinforce the invasive FLS phenotype; however, direct functional demonstration of an FLS-sEV-driven NF-κB-dependent macrophage program, including EV-mediated engagement of the miR-155–SHIP1 axis in recipient macrophages, is still required in RA. To be explicit about evidence level: FLS-derived sEV promotion of macrophage migration (8) and macrophage-derived sEV-driven RA-FLS proliferation via miRNA transfer (13) are directly demonstrated; the coordinated, NF-κB-dependent reciprocal circuit summarized above remains a hypothesis rather than an established mechanism in human RA. This reciprocal exchange between stromal and immune compartments may help sustain synovitis after upstream cytokine blockade and may partly explain residual disease activity that is not well captured by current clinical biomarkers. This mini-review examines EV-mediated communication in RA, with particular attention to mechanistic pathways, clinical heterogeneity, and the methodological and translational limitations that must be considered when interpreting this rapidly evolving field.

The relationship between EV-mediated signaling and canonical soluble cytokine pathways in RA is not yet resolved. At least three non-exclusive models can be distinguished. EV cargo could act largely downstream of TNF and IL-6 signaling, whereby cytokine-driven cellular activation states determine EV cargo composition. Under this model, effective cytokine blockade should eventually reduce pathogenic EV output.

Alternatively, EV signaling could operate largely in parallel with soluble cytokines. It may use shared downstream pathways such as NF-κB, MAPK, and JAK-STAT while being delivered through a spatially restricted particulate route that cytokine blockade does not directly interrupt. This model is consistent with the clinical observation that ultrasound-detected synovitis can persist despite normalized CRP and adequate cytokine-pathway inhibition (4).

A third possibility is that EV-associated cargo, including citrullinated autoantigens and other damage-associated molecules, sustains a genuinely cytokine-independent axis of immune activation. These models are not mutually exclusive and cannot yet be distinguished with existing data. Only the latter two predict that EV-directed biomarkers or therapeutics would add value beyond adequate cytokine-pathway blockade. Prospective studies pairing EV profiling with cytokine-pathway inhibitor response would help test this directly.

## 2 Extracellular vesicle biology and immune communication in RA

### 2.1 Biogenesis, cargo, and contamination issues

EVs are classified using biogenesis, particle size, density, surface markers, and isolation method, but these features do not always identify the same populations (5, 14). Classical sEVs form by inward budding of late endosomal membranes into multivesicular bodies (MVBs), which then fuse with the plasma membrane. Cellular activation, hypoxia, and metabolic stress can modify this ESCRT-dependent process (14). Microvesicles bud directly from the plasma membrane, whereas apoptotic bodies are released during programmed cell death. The distinction matters in RA studies: a platelet-enriched preparation and an FLS-enriched preparation may contain biologically different material, so incorrect assignment of EV origin can alter the mechanistic conclusion (5).

Synovial fluid makes EV isolation especially difficult (5, 15). It is viscous and protein-rich and contains lipoproteins, immune complexes, free ribonucleoproteins, and cellular debris, several of which co-sediment with EVs during ultracentrifugation. Isolation method, particle-to-protein ratio, and non-vesicular material can all change the apparent EV yield, protein concentration, and biological activity (15). Functional effects should therefore be assigned to EV cargo only after controls have excluded soluble proteins and other co-isolated material (15). Such controls are still inconsistent across RA studies; EV depletion by size-exclusion chromatography followed by proteinase K treatment, for example, is often absent. Stronger mechanistic claims will require MISEV2023-compliant characterization that combines particle tracking, orthogonal imaging, particle-to-protein ratios, and explicit contaminant controls.

Studies using better-characterized preparations have identified cargo with a credible role in RA biology. Synovial exosome preparations contain citrullinated fibrinogen and other citrullinated proteins that may function as autoantigens (16). Reported surface components include MHC class II molecules, the costimulatory ligands CD80 and CD86, complement opsonins, and integrins that may affect cellular tropism. Synovial-fluid EVs also contain miR-155, miR-146a, miR-21, and miR-223 (10). MiR-155 is of particular interest because it is associated with NF-κB-dependent inflammatory activation and macrophage polarization (11, 12), is repeatedly increased in RA synovial tissue, and contributes to disease severity in mouse collagen-induced arthritis (11, 12). The cargo profile is therefore compatible with active inflammatory programming, provided that non-vesicular RNA and protein have been adequately excluded.

### 2.2 EV uptake, synovial barrier penetration, and signaling

Recipient cells interact with EVs through surface receptor binding, clathrin-mediated endocytosis, macropinocytosis, membrane fusion, and phagocytic uptake (17). In the joint, these processes occur within viscoelastic synovial fluid rather than a freely diffusible medium. Hyaluronan concentrations are typically about 2–3 mg/mL in healthy joints but can fall below 1 mg/mL in inflamed joints as hyaluronidase activity increases and viscosity declines (18). This change is likely to affect EV transport. Neutrophil-derived microvesicles can enter cartilage in inflammatory arthritis, showing that vesicles of an appropriate size can cross the joint matrix and reach chondrocytes (19). Smaller sEVs (<200 nm) may move through the hyaluronan meshwork more readily than microvesicles larger than 500 nm, which may remain closer to synovial or perisynovial regions. Human RA tissue has not yet been mapped at sufficient resolution to confirm this distribution, so the proposed size effect should be treated as a biophysical expectation rather than an established spatial rule.

EV transport must also be considered in relation to synovial anatomy. The lining layer, sublining region, perivascular space, and cartilage-pannus junction contain different combinations of immune cells, stromal cells, and extracellular matrix. Patterns of EV release, retention, and uptake are therefore unlikely to be uniform across these sites. One working model is that sEVs released by sublining fibroblasts are taken up by lining macrophages, while macrophage-derived sEVs reaching the perivascular space influence endothelial adhesion molecule expression without requiring the parent cells to migrate. Such a relay could distribute inflammatory signals when cell trafficking is partly suppressed. It remains a model, however, because EV movement between these compartments has not been visualized directly in human RA synovium. To make the evidentiary status explicit, the synovial compartments and the size-dependent hyaluronan transport principles described above are established anatomical and biophysical features; the lining-to-sublining and macrophage-to-endothelium relay itself remains a hypothetical model that should be read as a proposed framework for future imaging studies rather than a demonstrated mechanism.

**Figure 1:**
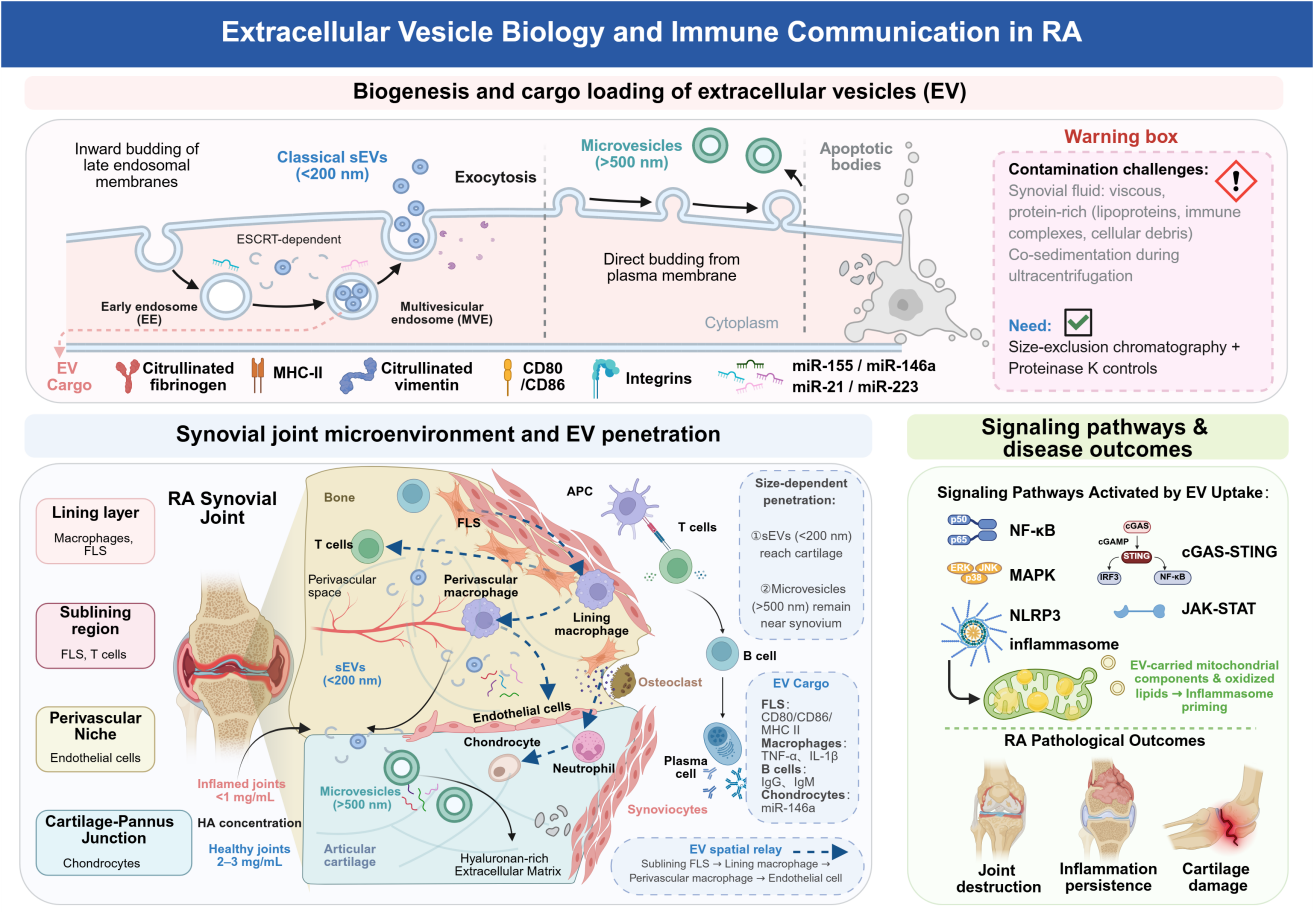
Extracellular vesicle biology and immune communication in rheumatoid arthritis.

EV uptake can activate inflammatory pathways such as NF-κB and MAPK in recipient cells, while mitochondrial danger signals are established activators of the NLRP3 inflammasome (20, 21). Direct evidence that EVs coordinately activate NF-κB, MAPK, JAK-STAT, cGAS-STING, and NLRP3 in intact human RA synovium is still lacking (**Figure 1**).

## 3 Pathogenic mechanisms of EV-mediated communication in RA

### 3.1 Synovial inflammation, innate immunity, and disease heterogeneity

Single-cell RNA sequencing data from the AMP RA consortium indicate that RA synovium is not a single tissue type, but rather comprises a set of discrete immune states (22). The lymphoid-myeloid pathotype is characterized by HLA-DR-high sublining macrophages, T-cell and B-cell infiltration, and ectopic lymphoid structures; its clinical behavior differs from that of the pauci-immune or fibroblast-dominant pathotypes, the latter showing expansion of CD34-negative FLS with minimal immune-cell infiltration. Macrophage composition also differs between active disease and sustained remission, with remission-associated MerTK-positive subsets linked to tissue repair and flare risk (23). EV profiling has not yet been systematically performed across these categories, but a pathotype-specific organization of EV communication can be hypothesized: in fibroblast-dominant disease, FLS-derived sEVs carrying invasive and matrix-remodeling cargo might constitute a dominant communication axis; in lymphoid-myeloid disease, dendritic cell- and macrophage-derived sEVs carrying citrullinated peptides and costimulatory signals may be more prominent. These remain testable hypotheses rather than established findings. Whether CD34-negative FLS can remotely influence myeloid progenitors in the bone marrow through circulating EVs, and thereby sustain systemic innate immune activation, remains an unresolved question with clear therapeutic implications.

Higher-resolution single-cell and treatment-response datasets refine this pathotype framework further. In synovial fluid profiled before and after TNF or JAK inhibitor therapy, SPP1+ macrophages show extensive predicted communication with CXCL13+CD4+ T cells, and this interaction is diminished in patients who respond to treatment (24); separately, low-input single-cell RNA sequencing of RA synovial tissue showed that distinct lining and sublining fibroblast subsets can be resolved from small clinical biopsies (25). Inflammatory myeloid states, including HLA-DR-expressing macrophage populations, have been resolved in larger RA single-cell studies (22, 23). Together, these findings support the feasibility of pairing single-cell pathotyping with paired EV sampling in prospective cohorts. Mapping candidate EV sources and recipients onto these defined populations remains hypothetical, but a concrete working assignment can be stated: SPP1+ and HLA-DR-high sublining macrophages are candidate sources of pro-inflammatory, citrullinated-antigen-carrying sEVs; CXCL13+CD4+ T cells and B cells within ectopic lymphoid aggregates are candidate recipients of, and possibly also contributors to, this vesicular signal; and CD34-negative pathogenic fibroblast subsets are candidates for both FLS-derived invasive EV cargo and for receiving reciprocal macrophage-derived signals. None of these source-recipient assignments has yet been tested with EV-specific single-cell or spatial methods; we present them as a concrete framework for hypothesis-driven validation rather than as established findings.

Platelet-derived microvesicles are abundant in inflamed synovial fluid and can stimulate synovial fibroblast inflammatory responses (7). Platelets and their microparticles nevertheless contribute through distinct mechanisms: GPVI (glycoprotein VI)-dependent platelet activation drives microparticle generation (7), whereas platelet COX-1-dependent prostacyclin production can promote synovitis independently of microparticle generation (26). Complement activation and complement receptors are established amplifiers of inflammatory and autoimmune disease (27), but the extent to which complement-opsonized EVs specifically drive RA synovitis remains to be directly established. We flag this distinction explicitly because complement involvement in RA overall is well established, whereas a specific role for complement-opsonized EVs is, at present, an extrapolation from general complement biology rather than a mechanism directly tested in synovial tissue.

Neutrophil-derived EVs and neutrophil extracellular trap (NET)-associated material appear to deliver a distinct set of inflammatory signals. NETs from RA patients contain citrullinated autoantigens and can augment inflammatory responses in synovial fibroblasts (28). More broadly, NET-associated nucleic acids and proteins can license macrophage cytokine production and support Th17 amplification (29). These findings support a plausible, although still incompletely proven, link between neutrophil death within the joint cavity, vesicular or NET-associated danger signals, and downstream inflammatory circuits relevant to anti-citrullinated protein antibody (ACPA)-positive disease.

### 3.2 Adaptive immunity, autoantigen presentation, and loss of immune tolerance

Processing of particulate antigens differs from that of soluble antigens. EV-associated antigens are more readily taken up by dendritic cells, often together with danger signals such as TLR ligands contained within the EV lumen, and may be cross-presented through both MHC class I and MHC class II pathways (30, 31). In this context, sEVs carrying citrullinated fibrinogen or other citrullinated proteins together with nucleic acid TLR ligands may provide a structural and immunological format that is more favorable for adaptive immune priming than soluble citrullinated proteins alone (30, 31). Whether this advantage is sufficient to amplify autoimmunity in established human RA, rather than mainly in well-controlled mouse models, remains uncertain because longitudinal human data are still limited.

Among EV-associated miRNAs in RA synovial fluid, miR-155 and miR-146a are among the best studied regulatory candidates (10). Functionally, miR-155 promotes inflammatory macrophage activation by suppressing SHIP1 and contributes to T helper 17 (Th17)-linked inflammatory arthritis programs (11, 12). By contrast, miR-146a can inhibit osteoclastogenesis and reduce joint destruction in collagen-induced arthritis models (32). This pattern suggests that regulatory miRNA signals are not completely absent in RA, but may be quantitatively or contextually outweighed by inflammatory cargo. Within this adaptive compartment, ectopic lymphoid structures resembling germinal centers are detected in approximately 25% of RA synovial biopsy specimens (33), and although EV-mediated antigen delivery offers a plausible mechanism for local autoantibody amplification, direct histological and functional evidence for B-cell-derived EVs within these structures in human RA tissue is still lacking.

### 3.3 FLS invasion, cartilage damage, bone erosion, and hypoxic remodeling

Activated FLS are major producers of matrix-degrading enzymes, including MMP-1, MMP-3, MMP-13, and ADAMTS family members (34). Proteomic characterization of size-exclusion-purified RA synovial-fluid EVs has identified an immunogenic, inflammation-associated cargo that includes matrix metalloproteinases such as MMP-9 (35), and FLS-derived sEVs in synovial fluid carry miRNA cargo capable of modulating inflammatory and stromal programs (10). Functionally, immune-cell-derived microparticles can induce MMP-1 and other MMP transcripts in cultured synovial fibroblasts (36), indicating that vesicular signals are sufficient to propagate matrix-degradation programs between cells. It nonetheless remains to be shown by dedicated proteomic or transcriptomic profiling that this full set of matrix-degrading enzymes and VEGF transcripts is selectively enriched within FLS-derived sEV preparations specifically. Recent work shows that RA synovial fibroblast-derived sEVs can be taken up by chondrocytes and induce chondrocyte apoptosis, with sEV-miRNA15–29148 proposed as a functional cargo (37). Whether the efficient miRNA transfer observed in monolayer coculture accurately reproduces the *in vivo* viscoelastic synovial fluid microenvironment requires validation in three-dimensional cartilage explant systems, because *in vivo* EV diffusion is restricted and cellular uptake competes with matrix trapping.

RANKL and RANK are functional components of EV-mediated communication in bone remodeling, particularly across osteoblast- or osteocyte-osteoclast signaling axes (38). Whether analogous RANKL-bearing EV pathways operate in RA synovium or contribute materially to periarticular erosion remains unknown. The anti-RANKL therapeutic denosumab reduces radiographic erosion scores, particularly erosion progression, but does not fully address joint-space narrowing or inflammatory disease activity (39).

The marked hypoxia of invasive synovial pannus is a powerful metabolic driver that alters FLS behavior (40). Under hypoxic stress, synovial fibroblast-derived exosomes can aggravate inflammatory imbalance, including a regulatory T cell (Treg)/Th17 shift; RA-FLS-derived EVs have also been shown to participate in angiogenic signaling through miRNA cargo (41, 42). Taken together with the invasive and matrix-remodeling functions described above, these findings suggest that hypoxia-conditioned EV biogenesis represents an additional layer of the same FLS-driven destructive program, linking the metabolic state of the pannus to angiogenesis and to the Treg/Th17 imbalance that sustains synovitis and structural damage (**Table 1**).

**Table 1:** Pathogenic mechanisms of EV-mediated communication in rheumatoid arthritis.

Pathogenic dimension	Confirmed EV evidence and related particulate findings	Evidence source	Major cellular targets	Proposed/hypothesized functional significance in RA
Innate inflammatory amplification	Confirmed EV evidence: platelet-derived, GPVI-dependent microparticles (7). Related non-vesicular evidence: NET-associated citrullinated autoantigens and extracellular DNA (28), and NET-associated nucleic acids/proteins that license macrophage cytokine production (29).	Human synovial fluid/platelets, ex vivo functional assay (7). Human RA neutrophil NETs plus *in vitro* synovial fibroblast stimulation (28). Animal model (murine atherosclerosis, not RA-specific) (29).	Demonstrated targets include synovial fibroblasts and macrophages; neutrophil, plasmacytoid dendritic-cell, and complement/Fc-receptor pathways remain putative components of the integrated circuit.	EVs are proposed to amplify synovial inflammation by linking platelet microparticle signaling, NET-associated danger signals, and myeloid-cell activation into one circuit that maintains inflammation beyond soluble cytokine signaling; this integrated circuit has not been directly demonstrated in RA synovium.
Adaptive immune priming and loss of tolerance	Citrullinated fibrinogen and other citrullinated proteins associated with synovial exosomes (16); antigen-presenting vesicle function of B lymphocytes (30); greater immunogenicity of dendritic-cell-derived exosomes versus microvesicles (31).	Human synovial fluid/tissue, association study (16). *In vitro*/animal B-cell and dendritic-cell models, not RA-specific (30, 31).	Dendritic cells, T cells, B cells, and ectopic lymphoid synovial aggregates	EVs are proposed to provide a particulate format for autoantigen delivery that supports anti-citrullinated immune responses and local adaptive amplification in RA synovium; this extrapolates from non-RA antigen-presentation models and has not been directly confirmed in RA tissue.
Stromal invasion and structural damage	RA synovial-fluid EV miRNA cargo, including miR-155, miR-146a, miR-21, and miR-223 (10); FLS-derived sEV induction of chondrocyte apoptosis via miRNA15–29148 (37).	Human RA synovial fluid (10). *In vitro* RA-FLS-chondrocyte coculture (37). RANKL/RANK EV biology and MMP/ADAMTS pathobiology drawn largely from general FLS/bone-remodeling literature, not RA-EV-specific tissue data (34, 38).	FLS, chondrocytes, osteoclast precursors, and the cartilage-pannus interface	EV-mediated stromal communication is proposed to promote pannus invasion, cartilage degradation, and periarticular bone erosion; the full matrix-degrading enzyme program and a RANKL-EV-mediated erosion pathway remain to be confirmed directly in RA synovial tissue.
Hypoxia and spatial niche remodeling	Hypoxia-conditioned synovial fibroblast exosome effects on Treg/Th17 balance (41); FLS-EV miRNA-1972-mediated angiogenic signaling to endothelial cells (42).	*In vitro*/animal (murine collagen-induced arthritis and hypoxic FLS culture) (41). *In vitro* endothelial coculture (42). General hypoxia-pannus biology drawn from a narrative review (40).	Demonstrated targets include T cells and endothelial cells; effects on macrophages, chondrocytes, and other perivascular synovial cells remain putative.	EVs are proposed to transmit hypoxic and metabolic programs across synovial microregions, promoting angiogenesis and niche-specific persistence of inflammation in human RA joints; direct *in situ* confirmation within intact hypoxic pannus is still lacking.

EV, extracellular vesicle; sEV, small extracellular vesicle; FLS, fibroblast-like synoviocyte; RA, rheumatoid arthritis; MMP, matrix metalloproteinase; ADAMTS, a disintegrin and metalloproteinase with thrombospondin motifs; RANKL, receptor activator of nuclear factor-κB ligand; NET, neutrophil extracellular trap; GPVI, glycoprotein VI; miRNA, microRNA; Fc, fragment crystallizable; Treg, regulatory T cell; Th17, T helper 17 cell.

## 4 Biomarkers, therapeutic potential, and clinical translation

### 4.1 Biomarker challenges and stratification opportunities

The conceptual appeal of EV biomarkers in RA lies in their potential tissue specificity. Macrophage-derived sEVs carrying M1-associated cargo and platelet-derived microvesicles expressing P-selectin convey different biological information even when particle concentrations are identical. Current composite measures, including CRP, ESR, DAS28, and ultrasound power Doppler, provide useful information on overall disease activity, but they are not designed to resolve the cellular source of persistent inflammation. In principle, EV-based signatures could help fill this gap. For example, a profile combining FLS-enriched markers with inflammatory EV cargo might eventually help identify fibroblast-dominant synovitis, but such combinations remain hypothetical and require validation in human RA (43, 44).

Several practical barriers, however, limit this possibility at present. In blood, abundant platelet-derived particles and non-vesicular contaminants can obscure low-abundance FLS- and leukocyte-derived sEV populations. Detecting these minor populations usually requires single-vesicle analysis or immunoaffinity enrichment, including aptamer-based capture and detection platforms (45), while preanalytical variation can alter EV yield, purity, and apparent cargo composition (5, 15, 46). Integrated lipidomic and multi-omic profiling has shown promise in arthritis biomarker research, although much of the supporting evidence remains preclinical or derives from non-human joint disease (44). For EV biomarkers to become clinically useful, they will need to outperform existing tools or add independent value beyond combinations of CRP, autoantibody titers, and ultrasound synovitis scores.

For RA specimens specifically, several pre-analytical variables are rarely standardized and can each distort apparent EV cargo independently of true biological differences. Synovial fluid viscosity and hyaluronan content affect particle recovery during centrifugation-based isolation (15); residual platelets and platelet-derived particles can dominate blood-derived EV counts and may also confound synovial-fluid EV measurements (5, 7, 15). Repeated freeze-thaw cycles, particularly when platelet depletion is incomplete, can alter EV recovery, subpopulation profiles, and apparent RNA cargo. Anticoagulant choice (citrate, EDTA, or heparin) can also alter platelet activation, complement activity, and downstream measurements ex vivo (5, 15). A further unresolved problem is that circulating and synovial-fluid miRNA is only partly vesicle-encapsulated: a substantial fraction travels bound to Argonaute-2 protein complexes or within lipoprotein particles, and most published RA EV-miRNA studies do not include the RNase-protection or lipoprotein-depletion controls needed to confirm that the measured signal is genuinely vesicular (5, 15). Until these pre-analytical variables are reported and controlled as standard practice, cross-study comparisons of RA EV biomarker candidates should be interpreted cautiously.

Single-particle flow cytometry can link marker expression with measurements at the level of individual EVs rather than averaged EV populations (46). Complementary imaging and affinity-based approaches are also emerging, although their scalability and cross-platform standardization remain unresolved. Looking ahead, spatial transcriptomics combined with single-vesicle imaging in frozen synovial sections could make it possible to map EV exchange within intact tissue architecture and define which synovial niches produce or receive specific sEV subsets. These approaches are not yet scalable for clinical use, but they point to the direction the field needs to move: from descriptive EV catalogs toward biomarkers and tools grounded in disease mechanism.

### 4.2 Therapeutic potential and translational limitations

Mesenchymal stem cell (MSC)-derived sEVs have the strongest therapeutic rationale among EV populations, but the specific therapeutic claims should be separated by level of evidence. In inflammatory arthritis models, MSC-derived exosomes are more immunosuppressive than the corresponding microparticles, contain measurable immunomodulatory mediators such as TGF-β1, and attenuate collagen-induced arthritis with associated changes in lymphocyte and B-cell compartments (47). Complementing these animal data, MSC-EVs, particularly after IFN-β priming, have shown immunomodulatory effects on RA CD4+ T cells and inhibition of RA-FLS migration ex vivo (48). Clinical translation nonetheless requires standardized source materials, potency assays, biodistribution, target engagement, and reproducible pharmacodynamic endpoints (43). Human umbilical-cord MSC-derived sEVs have also been reported to ameliorate collagen-induced arthritis through immunomodulatory effects on T lymphocytes (49), while MSC-sEVs can directly suppress Th17 biology by destabilizing RORγt in a defined autoimmune inflammation model (50). For macrophage-directed effects, bone marrow MSC-derived exosomes carrying miR-223 have been reported to suppress NLRP3 activation and inflammatory cytokine release in macrophages and to ameliorate disease in experimental RA (51).

Engineered exosome systems, including exosomes carrying a super-repressor of NF-κB, have shown proof-of-concept anti-inflammatory activity and joint protection in preclinical RA models (52). Their manufacturing pathway is far more complex than that of recombinant biologics. Batch-to-batch consistency in potency defined per biologically active particle, miRNA cargo, and particle size distribution has not yet been solved at GMP scale for any sEV therapeutic product. Off-target hepatic and splenic sequestration remains a pharmacokinetic challenge. Current translational limitations suggest that stand-alone sEV immunotherapies are unlikely to replace biologics in the near term; a more feasible near-term application is to use sEVs for local delivery of small-molecule or RNA cargo to inflamed synovium, thereby reducing systemic exposure relative to intravenous administration.

Placed alongside conventional biologic and targeted synthetic DMARDs, the translational gap for sEV therapeutics becomes clearer across several practical dimensions.

Manufacturability: biologics and small-molecule targeted synthetic DMARDs are produced from well-characterized cell lines or by chemical synthesis with validated release criteria. By contrast, sEV production depends on primary or stem-cell culture conditions that can shift vesicle yield and cargo between batches and manufacturing sites. Potency assessment: biologic potency is supported by validated, target-specific functional assays and established release criteria. For sEVs, potency would need to be linked to a defined biological activity, particle dose, and relevant cargo attributes; no standardized potency framework has yet been established for an RA-directed sEV product.

Biodistribution and safety monitoring: monoclonal antibodies and small molecules have extensive pharmacokinetic, immunogenicity, and postmarketing safety data. sEVs are cleared rapidly by the liver and spleen through incompletely characterized mechanisms, joint-specific accumulation after systemic administration has not been demonstrated in humans, and comparable long-term human safety data for RA-directed sEV products do not yet exist. These comparisons show that sEV-based therapeutics for RA remain less mature than approved biologic or targeted synthetic agents; claims of near-term clinical readiness should therefore be tempered.

## 5 Discussion and future perspectives

To date, the most productive contribution of EV biology to RA has been the hypothesized positive-feedback communication model: FLS may reprogram macrophages through sEV cargo, and macrophages may in turn reprogram FLS. This reciprocal exchange could help maintain synovitis when soluble cytokine activity is suppressed, but it has not been directly demonstrated as a cytokine-independent mechanism in human RA. This mechanism is consistent with clinical observations that ultrasound-detected synovitis may persist after normalization of serum inflammatory markers (4), and with therapeutic data showing that erosion progression and inflammatory disease activity can be partially dissociated (39). Whether EV-mediated intercellular communication is a primary driver of this residual disease or a secondary amplifier of cytokine-primed cellular activation remains the key mechanistic question and is still unanswered.

The next experiments should connect EV measurements to tissue biology. Longitudinal clinical studies could pair EV profiling with synovial biopsy, imaging, and treatment response to test whether sEV cargo identifies macrophage or FLS activation more accurately than serum CRP. Spatial analysis of intact RA synovium could then determine how established molecular pathotypes (53), including newer single-cell and treatment-response atlases (24, 25), relate to the cells that release and receive specific sEV subsets; single-vesicle methods provide a technical starting point (46). Mechanistic work also needs compositionally defined, contamination-controlled sEV preparations tested in three-dimensional FLS-macrophage cocultures and cartilage explants. Dose-response experiments should use particle concentrations that are plausible in human synovial fluid.

These studies may also clarify whether EV profiles have value for treatment stratification. Lymphoid-myeloid and fibroblast-dominant RA could have different patterns of sEV exchange, making synovial-fluid EVs or immunoaffinity-captured circulating subsets potentially useful before biologic selection (44, 53). One testable example would compare patients with a high miR-155/miR-146a sEV ratio and cadherin-11-positive circulating sEVs with those whose synovial fluid contains predominantly complement-opsonized platelet EVs. No such framework is ready for clinical use, but it defines a specific hypothesis for prospective validation.

EVs are part of the communication network that links inflammation, stromal activation, and tissue damage in the rheumatoid joint. Their clinical value remains uncertain because most studies are descriptive, use heterogeneous isolation methods, or lack direct tissue validation. Progress now depends on contamination-controlled mechanistic experiments, spatial analysis of synovium, harmonized preanalytical protocols, and prospective EV measurements embedded in RA clinical trials.

## References

[1] Smolen JS Aletaha D Barton A Burmester GR Emery P Firestein GS . Rheumatoid arthritis. Nat Rev Dis Primers. (2018) 4:18001. doi: 10.1016/b978-0-443-23947-2.00081-3 29417936

[2] Burmester GR Pope JE . Novel treatment strategies in rheumatoid arthritis. Lancet. (2017) 389:2338–48. doi: 10.1016/s0140-6736(17)31491-5 28612748

[3] Sung YK Yoshida K Prince FHM Frits ML Cho SK Choe JY . Prevalence and predictors for sustained remission in rheumatoid arthritis. PloS One. (2019) 14:e0214981. doi: 10.1371/journal.pone.0214981 31002669 PMC6474583

[4] Nguyen H Ruyssen-Witrand A Gandjbakhch F Constantin A Foltz V Cantagrel A . Prevalence of ultrasound-detected residual synovitis and risk of relapse and structural progression in rheumatoid arthritis patients in clinical remission: a systematic review and meta-analysis. Rheumatology. (2014) 53:2110–8. doi: 10.1093/rheumatology/keu217 24929634

[5] Welsh JA Goberdhan DCI O’Driscoll L Buzas EI Blenkiron C Bussolati B . Minimal information for studies of extracellular vesicles (MISEV2023): from basic to advanced approaches. J Extracell Vesicles. (2024) 13:e12404. doi: 10.1002/jev2.12404 38326288 PMC10850029

[6] van Niel G D’Angelo G Raposo G . Shedding light on the cell biology of extracellular vesicles. Nat Rev Mol Cell Biol. (2018) 19:213–28. doi: 10.1038/nrm.2017.125 29339798

[7] Boilard E Nigrovic PA Larabee K Watts GFM Coblyn JS Weinblatt ME . Platelets amplify inflammation in arthritis via collagen-dependent microparticle production. Science. (2010) 327:580–3. doi: 10.1126/science.1181928 20110505 PMC2927861

[8] Nakamachi Y Uto K Hayashi S Okano T Morinobu A Kuroda R . Exosomes derived from synovial fibroblasts from patients with rheumatoid arthritis promote macrophage migration that can be suppressed by miR-124-3p. Heliyon. (2023) 9:e14986. doi: 10.1016/j.heliyon.2023.e14986 37151687 PMC10161379

[9] Stanczyk J Pedrioli DML Brentano F Sanchez-Pernaute O Kolling C Gay RE . Altered expression of microRNA in synovial fibroblasts and synovial tissue in rheumatoid arthritis. Arthritis Rheum. (2008) 58:1001–9. doi: 10.1002/art.23386 18383392

[10] Foers AD Garnham AL Chatfield S Smyth GK Cheng L Hill AF . Extracellular vesicles in synovial fluid from rheumatoid arthritis patients contain miRNAs with capacity to modulate inflammation. Int J Mol Sci. (2021) 22:4910. doi: 10.3390/ijms22094910 34066338 PMC8125513

[11] Kurowska-Stolarska M Alivernini S Ballantine LE Asquith DL Millar NL Gilchrist DS . MicroRNA-155 as a proinflammatory regulator in clinical and experimental arthritis. Proc Natl Acad Sci USA. (2011) 108:11193–8. doi: 10.1073/pnas.1019536108 21690378 PMC3131377

[12] Blüml S Bonelli M Niederreiter B Puchner A Mayr G Hayer S . Essential role of microRNA-155 in the pathogenesis of autoimmune arthritis in mice. Arthritis Rheum. (2011) 63:1281–8. doi: 10.1002/art.30281 21321928

[13] Liu H Chen Y Huang Y Wei L Ran J Li Q . Macrophage-derived miR-100-5p orchestrates synovial proliferation and inflammation in rheumatoid arthritis through mTOR signaling. J Nanobiotechnol. (2024) 22:197. doi: 10.1186/s12951-024-02444-1 38644475 PMC11034106

[14] Colombo M Raposo G Théry C . Biogenesis, secretion, and intercellular interactions of exosomes and other extracellular vesicles. Annu Rev Cell Dev Biol. (2014) 30:255–89. doi: 10.1146/annurev-cellbio-101512-122326 25288114

[15] Coumans FAW Brisson AR Buzas EI Dignat-George F Drees EEE El-Andaloussi S . Methodological guidelines to study extracellular vesicles. Circ Res. (2017) 120:1632–48. doi: 10.1161/circresaha.117.309417 28495994

[16] Skriner K Adolph K Jungblut PR Burmester GR . Association of citrullinated proteins with synovial exosomes. Arthritis Rheum. (2006) 54:3809–14. doi: 10.1002/art.22276 17133577

[17] Mulcahy LA Pink RC Carter DRF . Routes and mechanisms of extracellular vesicle uptake. J Extracell Vesicles. (2014) 3:24641. doi: 10.3402/jev.v3.24641 25143819 PMC4122821

[18] Dahl LB Dahl IMS Engström-Laurent A Granath K . Concentration and molecular weight of sodium hyaluronate in synovial fluid from patients with rheumatoid arthritis and other arthropathies. Ann Rheum Dis. (1985) 44:817–22. doi: 10.1007/978-3-031-20882-9_12 4083937 PMC1001790

[19] Headland SE Jones HR Norling LV Kim A Souza PR Corsiero E . Neutrophil-derived microvesicles enter cartilage and protect the joint in inflammatory arthritis. Sci Transl Med. (2015) 7:315ra190. doi: 10.1126/scitranslmed.aac5608 26606969 PMC6034622

[20] Sanwlani R Gangoda L . Role of extracellular vesicles in cell death and inflammation. Cells. (2021) 10:2663. doi: 10.3390/cells10102663 34685643 PMC8534608

[21] Misawa T Takahama M Kozaki T Lee H Zou J Saitoh T . Microtubule-driven spatial arrangement of mitochondria promotes activation of the NLRP3 inflammasome. Nat Immunol. (2013) 14:454–60. doi: 10.1038/ni.2550 23502856

[22] Zhang F Wei K Slowikowski K Fonseka CY Rao DA Kelly S . Defining inflammatory cell states in rheumatoid arthritis joint synovial tissues by integrating single-cell transcriptomics and mass cytometry. Nat Immunol. (2019) 20:928–42. doi: 10.1038/s41590-019-0378-1 31061532 PMC6602051

[23] Alivernini S MacDonald L Elmesmari A Finlay S Tolusso B Gigante MR . Distinct synovial tissue macrophage subsets regulate inflammation and remission in rheumatoid arthritis. Nat Med. (2020) 26:1295–306. doi: 10.1038/s41591-020-0939-8 32601335

[24] Xia X He C Xue Z Wang Y Qin Y Ren Z . Single cell immunoprofile of synovial fluid in rheumatoid arthritis with TNF/JAK inhibitor treatment. Nat Commun. (2025) 16:2152. doi: 10.1038/s41467-025-57361-0 40038288 PMC11880340

[25] Stephenson W Donlin LT Butler A Rozo C Bracken B Rashidfarrokhi A . Single-cell RNA-seq of rheumatoid arthritis synovial tissue using low-cost microfluidic instrumentation. Nat Commun. (2018) 9:791. doi: 10.1038/s41467-017-02659-x 29476078 PMC5824814

[26] Boilard E Larabee K Shnayder R Jacobs K Farndale RW Ware J . Platelets participate in synovitis via Cox-1-dependent synthesis of prostacyclin independently of microparticle generation. J Immunol. (2011) 186:4361–8. doi: 10.1016/b978-0-323-31696-5.00016-4 21357261 PMC3208424

[27] Holers VM . Complement and its receptors: new insights into human disease. Annu Rev Immunol. (2014) 32:433–59. doi: 10.1146/annurev-immunol-032713-120154 24499275

[28] Khandpur R Carmona-Rivera C Vivekanandan-Giri A Gizinski A Yalavarthi S Knight JS . NETs are a source of citrullinated autoantigens and stimulate inflammatory responses in rheumatoid arthritis. Sci Transl Med. (2013) 5:178ra40. doi: 10.1126/scitranslmed.3005580 23536012 PMC3727661

[29] Warnatsch A Ioannou M Wang Q Papayannopoulos V . Neutrophil extracellular traps license macrophages for cytokine production in atherosclerosis. Science. (2015) 349:316–20. doi: 10.1126/science.aaa8064 26185250 PMC4854322

[30] Raposo G Nijman HW Stoorvogel W Liejendekker R Harding CV Melief CJM . B lymphocytes secrete antigen-presenting vesicles. J Exp Med. (1996) 183:1161–70. doi: 10.1084/jem.183.3.1161 8642258 PMC2192324

[31] Wahlund CJE Güclüler G Hiltbrunner S Veerman RE Näslund TI Gabrielsson S . Exosomes from antigen-pulsed dendritic cells induce stronger antigen-specific immune responses than microvesicles *in vivo*. Sci Rep. (2017) 7:17095. doi: 10.1038/s41598-017-16609-6 29213052 PMC5719080

[32] Nakasa T Shibuya H Nagata Y Niimoto T Ochi M . The inhibitory effect of microRNA-146a expression on bone destruction in collagen-induced arthritis. Arthritis Rheum. (2011) 63:1582–90. doi: 10.1002/art.30321 21425254

[33] Humby F Bombardieri M Manzo A Kelly S Blades MC Kirkham B . Ectopic lymphoid structures support ongoing production of class-switched autoantibodies in rheumatoid synovium. PloS Med. (2009) 6:e1. doi: 10.1371/journal.pmed.0060001 19143467 PMC2621263

[34] Huber LC Distler O Tarner I Gay RE Gay S Pap T . Synovial fibroblasts: key players in rheumatoid arthritis. Rheumatology. (2006) 45:669–74. doi: 10.1093/rheumatology/kel065 16567358

[35] Foers AD Dagley LF Chatfield S Webb AI Cheng L Hill AF . Proteomic analysis of extracellular vesicles reveals an immunogenic cargo in rheumatoid arthritis synovial fluid. Clin Transl Immunol. (2020) 9:e1185. doi: 10.1002/cti2.1185 33204424 PMC7648259

[36] Distler JHW Jüngel A Huber LC Seemayer CA Reich C Gay RE . The induction of matrix metalloproteinase and cytokine expression in synovial fibroblasts stimulated with immune cell microparticles. Proc Natl Acad Sci USA. (2005) 102:2892–7. doi: 10.1073/pnas.0409781102 15701693 PMC548330

[37] Zhang Z Liu L Ti H Chen M Chen Y Du D . Synovial fibroblast derived small extracellular vesicles miRNA15-29148 promotes articular chondrocyte apoptosis in rheumatoid arthritis. Bone Res. (2025) 13:61. doi: 10.1038/s41413-025-00430-3 40506465 PMC12162823

[38] Holliday LS Patel SS Rody WJ Jr . RANKL and RANK in extracellular vesicles: surprising new players in bone remodeling. Extracell Vesicles Circ Nucleic Acids. (2021) 2:18–28. doi: 10.20517/evcna.2020.02 33982033 PMC8112638

[39] Takeuchi T Tanaka Y Ishiguro N Yamanaka H Yoneda T Ohira T . Effects of the anti-RANKL antibody denosumab on joint structural damage in patients with rheumatoid arthritis treated with conventional synthetic disease-modifying antirheumatic drugs (DESIRABLE study): a randomised, double-blind, placebo-controlled phase 3 trial. Ann Rheum Dis. (2019) 78:899–907. doi: 10.1136/annrheumdis-2018-214827 31036625 PMC6585575

[40] Fearon U Canavan M Biniecka M Veale DJ . Hypoxia, mitochondrial dysfunction and synovial invasiveness in rheumatoid arthritis. Nat Rev Rheumatol. (2016) 12:385–97. doi: 10.1038/nrrheum.2016.69 27225300

[41] Ding Y Wang L Wu H Zhao Q Wu S . Exosomes derived from synovial fibroblasts under hypoxia aggravate rheumatoid arthritis by regulating Treg/Th17 balance. Exp Biol Med. (2020) 245:1177–86. doi: 10.1177/1535370220934736 32615822 PMC7437376

[42] Chen Y Dang J Lin X Wang M Liu Y Chen J . RA fibroblast-like synoviocytes derived extracellular vesicles promote angiogenesis by miRNA-1972 targeting p53/mTOR signaling in vascular endotheliocyte. Front Immunol. (2022) 13:793855. doi: 10.3389/fimmu.2022.793855 35350778 PMC8957937

[43] Cosenza S Ruiz M Maumus M Jorgensen C Noël D . Pathogenic or therapeutic extracellular vesicles in rheumatic diseases: role of mesenchymal stem cell-derived vesicles. Int J Mol Sci. (2017) 18:889. doi: 10.3390/ijms18040889 28441721 PMC5412468

[44] Varela L van de Lest CHA van Weeren PR Wauben MHM . Synovial fluid extracellular vesicles as arthritis biomarkers: the added value of lipid-profiling and integrated omics. Extracell Vesicles Circ Nucleic Acids. (2024) 5:276–96. doi: 10.20517/evcna.2024.14 39698533 PMC11648409

[45] Zhu L Tian W Yuan L Chi C Wang Y Xiao Q . Aptamer-based extracellular vesicle isolation, analysis and therapeutics. Interdiscip Med. (2023) 1:e20220019. doi: 10.1002/inmd.20220019 41531421

[46] Nolan JP Duggan E . Analysis of individual extracellular vesicles by flow cytometry. Methods Mol Biol. (2018) 1678:79–92. doi: 10.1007/978-1-4939-7346-0_5 29071676

[47] Cosenza S Toupet K Maumus M Luz-Crawford P Blanc-Brude O Jorgensen C . Mesenchymal stem cells-derived exosomes are more immunosuppressive than microparticles in inflammatory arthritis. Theranostics. (2018) 8:1399–410. doi: 10.7150/thno.21072 29507629 PMC5835945

[48] Tsiapalis D Floudas A Tertel T Boerger V Giebel B Veale DJ . Therapeutic effects of mesenchymal/stromal stem cells and their derived extracellular vesicles in rheumatoid arthritis. Stem Cells Transl Med. (2023) 12:849–62. doi: 10.1093/stcltm/szad065 37934808 PMC10726408

[49] Xu K Ma D Zhang G Gao J Su Y Liu S . Human umbilical cord mesenchymal stem cell-derived small extracellular vesicles ameliorate collagen-induced arthritis via immunomodulatory T lymphocytes. Mol Immunol. (2021) 135:36–44. doi: 10.1016/j.molimm.2021.04.001 33857817

[50] Jung S Lee S Kim HJ Kim S Moon JH Chung H . Mesenchymal stem cell-derived extracellular vesicles subvert Th17 cells by destabilizing RORγt through posttranslational modification. Exp Mol Med. (2023) 55:665–79. doi: 10.1038/s12276-023-00949-7 36964252 PMC10073130

[51] Huang Y Lu D Ma W Liu J Ning Q Tang F . miR-223 in exosomes from bone marrow mesenchymal stem cells ameliorates rheumatoid arthritis via downregulation of NLRP3 expression in macrophages. Mol Immunol. (2022) 143:68–76. doi: 10.1016/j.molimm.2022.01.002 35042119

[52] Lee HI Ahn MJ Yoo JK Ahn SH Park SY Seo H . Exosome-mediated delivery of super-repressor IkappaBalpha alleviates inflammation and joint damages in rheumatoid arthritis. Arthritis Res Ther. (2024) 26:2. doi: 10.1186/s13075-023-03225-1 38167497 PMC10759503

[53] Lewis MJ Barnes MR Blighe K Goldmann K Rana S Hackney JA . Molecular portraits of early rheumatoid arthritis identify clinical and treatment response phenotypes. Cell Rep. (2019) 28:2455–2470.e5. doi: 10.1016/j.celrep.2019.07.091 31461658 PMC6718830

